# Vesicle-associated membrane protein 7-mediated eosinophil degranulation promotes allergic airway inflammation in mice

**DOI:** 10.1038/s42003-018-0081-z

**Published:** 2018-06-29

**Authors:** Lian Willetts, Lindsey C. Felix, Elizabeth A. Jacobsen, Lakshmi Puttagunta, Rachel M. Condjella, Katie R. Zellner, Sergei I. Ochkur, John D. Kim, Huijun Luo, Nancy A. Lee, James J. Lee, Redwan Moqbel, Paige Lacy

**Affiliations:** 1 Alberta Respiratory Centre (ARC) Research, Department of Medicine, University of Alberta, Edmonton, T6G 2S2 Alberta Canada; 2 Division of Pulmonary Medicine, Department of Biochemistry and Molecular Biology, Mayo Clinic Arizona, Scottsdale, 85259 AZ USA; 3 Department of Laboratory Medicine & Pathology, University of Alberta, Edmonton, T6G 2B7 Alberta Canada; 4 Department of Immunology, University of Manitoba, Winnipeg, R3T 2N2 Manitoba Canada

## Abstract

Eosinophil degranulation is a determining factor in allergy-mediated airway pathology. Receptor-mediated degranulation in eosinophils requires vesicle-associated membrane protein 7 (VAMP-7), a principal component of the SNARE fusion machinery. The specific contribution of eosinophil degranulation to allergen-induced airway responses remains poorly understood. We generated mice with *VAMP-7* gene deficiency exclusively in eosinophils (*eoCRE/V7*) from a cross using eosinophil-specific *Cre* recombinase-expressing mice crossed with *VAMP-7*^*f/f*^ mice. Eosinophils from *eoCRE/V7* mice showed deficient degranulation responses in vitro, and responses continued to be decreased following ex vivo intratracheal adoptive transfer of *eoCRE/V7* eosinophils into *IL-5/hE2/EPX*^*−/−*^ mice. Consistent with diminished degranulation responses, reduced airway hyperresponsiveness was observed in ovalbumin-sensitized and challenged *eoCRE/V7* mice following methacholine inhalation. Therefore, VAMP-7 mediates eosinophil degranulation both in vitro and ex vivo, and this event augments airway hyperresponsiveness.

## Abstract (web summary)

Lian Willetts et al. demonstrate that vesicle-associated membrane protein 7 (VAMP 7), a principal component of the membrane fusion machinery, promotes eosinophil degranulation in allergic airway inflammation. This study suggests VAMP7 as a therapeutic target for ameliorating asthma.

## Introduction

Eosinophil activation is a major factor in exacerbation of asthma. Approximately 50% of all asthmatic subjects exhibit evidence of eosinophils in their airways^1^. Corticosteroids are the main treatment used in anti-inflammatory treatment of asthma and reduction in eosinophil levels, but these fail to ameliorate symptoms in all patients even at enhanced doses. Thus, a more tailored approach for intervention in asthma exacerbation is required to target underlying inflammatory events. This is evident from recent clinical trials showing that eosinophil depletion by monoclonal anti-IL-5 or IL-5 receptor antibody (e.g., mepolizumab, reslizumab, and benralizumab) leads to corticosteroid dose tapering and reduced exacerbations and diminished rates of hospitalization^2–4^.

In asthma, airway eosinophilia is a feature of atopic and severe non-atopic late-onset asthma. Eosinophils are considered predominantly pro-inflammatory, and contribute to tissue damage by the release of cytotoxic cationic granule proteins through degranulation^5^. Eosinophil degranulation is defined as regulated receptor-mediated release of granule products that serves to directly deposit secreted mediators toward a target^6^. Receptor-mediated degranulation in eosinophils in response to a range of inflammatory stimuli is dependent on the final fusion step occurring between secretory granules and the plasma membrane, known as regulated exocytosis^7^. This requires distal binding of membrane-bound fusion-competent soluble *N*-ethylmaleimide sensitive factor attachment protein (SNAP) receptors (SNAREs) on granule and cell membranes, which facilitate fusion of granules and the subsequent release of contents to cell exterior. SNAREs comprise core components of the fusion machinery necessary for exocytosis in all secretion-competent cells^8^, and are expressed in eosinophils^9–11^. Core components of SNARE complexes expressed by eosinophils include vesicle-associated membrane protein (VAMP) 2, 7, 8, syntaxin-4, and SNAP-23^11,12^. The R-SNARE VAMP-7 co-localizes with eosinophil peroxidase (EPX), an oxidoreductase that is uniquely expressed in eosinophils and stored within crystalloid granules^11,13^. VAMP-7 is required for agonist-activated release of EPX via degranulation from streptolysin-*O*-permeabilized human eosinophils^11^. However, no reports have demonstrated a causal link between physiologically induced eosinophil degranulation and the development of airway hyperresponsiveness (AHR) or airway remodeling. Here we sought to determine the role of VAMP-7-mediated eosinophil degranulation in allergic airway inflammation as an important exacerbatory event in asthma.

## Results

### VAMP-7 co-localizes with EPX-containing crystalloid granules

**Fig. 1:**
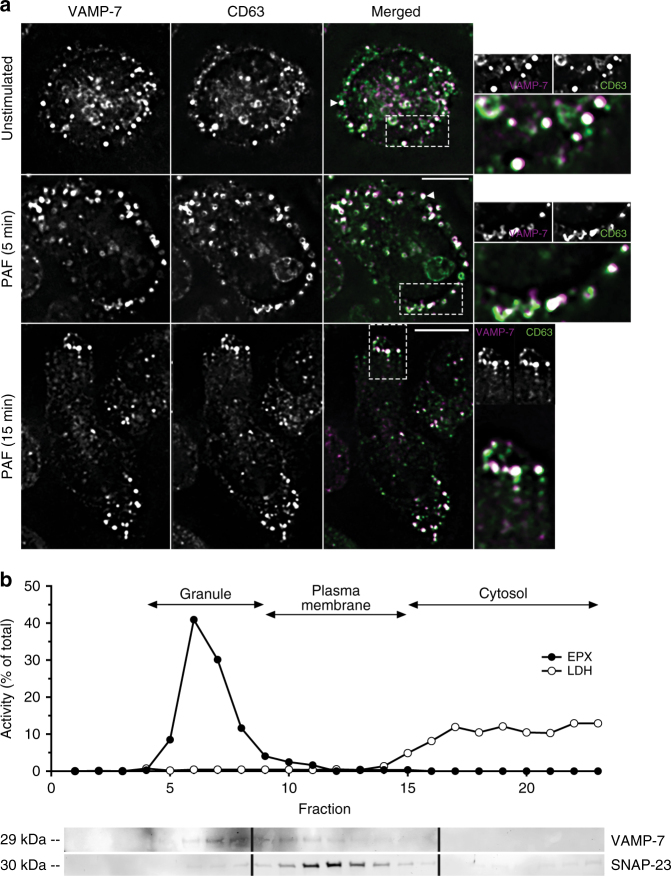
VAMP-7 expression and co-localization with crystalloid granules. **a** Unstimulated and PAF-stimulated (5 μM; 5 and 15 min) adherent eosinophils isolated from the peripheral blood of *I5* mice were fixed and stained for VAMP-7 and CD63 immunoreactivity. Arrowheads (white) indicate co-localization of VAMP-7 and CD63 to the membranes of crystalloid granules. Stimulated cells represented 30–40% of total population in 10 high-powered fields. Scale bar for merged PAF (5 min) applies to top and middle panels, 5 μm; scale bar for merged PAF (15 min) applies to lower panel only, 5 μm. Images at right are magnified from boxes shown in dotted lines. White indicates magenta-green overlap. **b** Subcellular fractionation of eosinophils (5 × 10^7^) isolated from peripheral blood of four *I5* mice followed by western blot analysis of fractions. In subcellular fractions, EPX was detected in fractions 5–9, and lactate dehydrogenase^+^ cytosolic fractions were enriched in fractions 15–23. Western blot analysis showed VAMP-7 concentrated in fractions 5–10, while SNAP-23 (cognate Q-SNARE for VAMP-7) was found in fractions 10–14. Representative of three independent experiments

To confirm mouse eosinophil expression of VAMP-7, immunofluorescence analysis was used to determine the co-localization of VAMP-7 with CD63, a marker for crystalloid granules^14^. Eosinophils were isolated from *IL-5* transgenic mice (*I5*) for immunofluorescence and subcellular fractionation because of their abundance in *I5* mice over wild-type strains. Immunofluorescence for CD63 revealed characteristic granule-associated punctate patterns of staining co-localizing with VAMP-7 (Pearson’s co-localization coefficient *r*^2^ = 0.45 ± 0.02) (Fig. 1a), indicating expression of VAMP-7 in crystalloid granules. Upon stimulation by platelet activating factor (PAF, 5 μM), both VAMP-7^+^ and CD63^+^ granules translocated to and co-localized at the cell periphery (*r*^2^ = 0.45 ± 0.04) of chemotaxing cells (Fig. 1a), suggestive of exocytotic release.

To confirm co-localization with granules, eosinophils were fractionated and separated on a linear density gradient (0–45%). Eosinophils showed enriched EPX activity in crystalloid granule-containing fractions that overlapped with peak VAMP-7 immunoreactivity (fractions 5–10; Fig. 1b and Supplementary Figure 1a) and less so with plasma membrane SNAP-23 (fractions 10–14), a cognate Q-SNARE binding partner for VAMP-7^9^.

### Eosinophil-specific *VAMP-7* gene deletion in *eoCRE/V7* mice

**Fig. 2:**
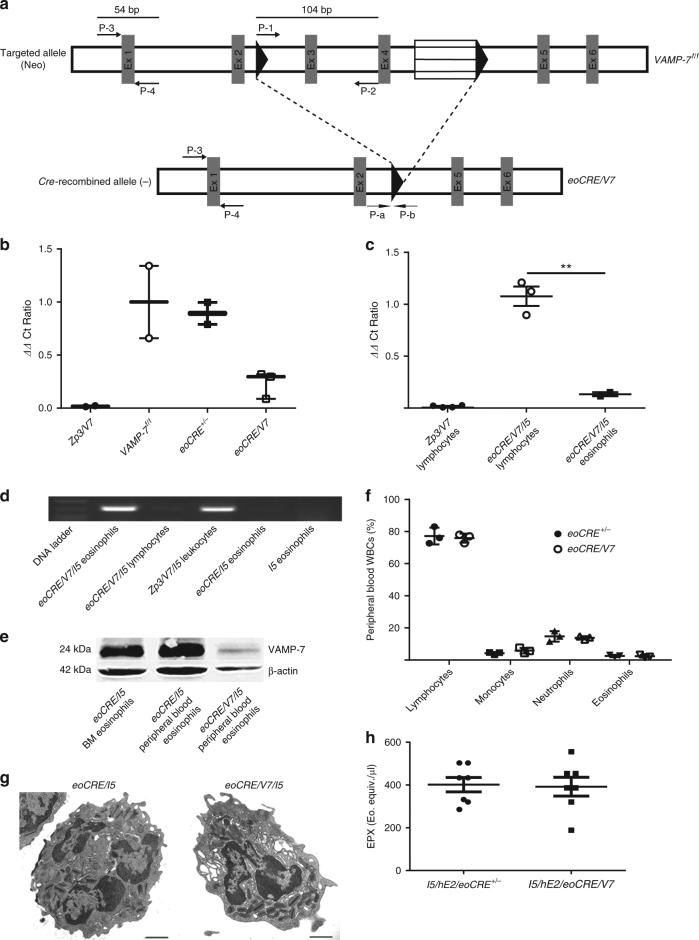
Eosinophil-specific *VAMP-7* gene deletion in *eoCRE/V7* mice confirmed by qPCR. **a** *VAMP-7* gene deletion protocol used to generate *eoCRE/V7* offspring. Amplicon values obtained from *eoCRE/V7* offspring were compared to control *VAMP-7*^*f/f*^ mice to generate ΔCt and ΔΔCt ratios as described in Methods section. Diagram adapted from Sato et al.^32^. **b** qPCR-quantified *VAMP-7* gene deletion in bone marrow-derived eosinophils isolated from *Zp3/V7*, *VAMP-7*^*f/f*^, *eoCRE*^*+/−*^, and *eoCRE/V7* mice. Low ΔΔCt values indicate *VAMP-7* gene deletion. All data were normalized to *VAMP-7*^*f/f*^ values. Data are presented as mean ± min-max range. **c** *VAMP-7* gene excision efficiency between eosinophils and lymphocytes isolated from *eoCRE/V7/I5* mice. Peripheral blood eosinophils (CCR3^+^/IL-5Rα^+^/Gr-1^+^/CD4^*−*^/B220^*−*^) and lymphocytes (CCR3^−^/IL-5Rα^*−*^/Gr-1^−^/CD4^+^/B220^+^) were analyzed from *Zp3/V7* or *eoCRE/V7/I5* mice. All data were normalized to the average ΔΔCt value for the *eoCRE/V7/I5* lymphocyte population. Closed symbols indicate *eoCRE*^*+/−*^ mice, open symbols indicate *eoCRE/V7* mice. **d** VAMP-7-specific gene deletion was restricted to *eoCRE/V7/I5*-derived eosinophils. The P-a and P-b primer pair shown in (**a**) was used to detect expression of the deleted gene sequence (326 bps) in both eosinophils (CCR3^+^/IL-5Rα^+^/Gr-1^+^/CD4^−^/B220^−^) and lymphocytes (CCR3^−^/IL-5Rα^*−*^/Gr-1^*−*^/CD4^+^/B220^+^) isolated from *eoCRE/V7/I5* mice. Expression profiles of the latter strain were compared to *Zp3/V7/I5* whole-blood leukocytes, *eoCRE/I5* eosinophils, and *I5* eosinophils. **e** Western blot analysis of VAMP-7 protein expression in *eoCRE/V7/I5*-derived eosinophils (~96% purity), as well as in bone marrow-and peripheral blood-derived eosinophils from eoCRE/I5 mice. Gel was cropped to improve conciseness of presentation. **f** peripheral blood WBC profiles of *eoCRE*^*+/−*^ and *eoCRE/V7* mice. **g** Transmission electron microscopy images of peripheral blood eosinophils from *eoCRE/I5* and *eoCRE/V7/I5* mice. Scale bar, 1 μm. **h** EPX levels in BAL fluid from *I5/hE2/eoCRE*^*+/−*^ and *I5/hE2/eoCRE/V7* mice. For Fig. 2b, c, f, and h, *n* = 3–7 mice/experiment. Data are presented as mean ± SEM. For Fig. 2d, e, and g, one representative gel, blot, or image from three separate experiments is shown. **p < 0.01 (one-way ANOVA, Tukey’s)

To investigate a possible role for VAMP-7 in eosinophil degranulation and allergic inflammation, we crossed mice expressing *Cre* recombinase from the eosinophil-specific EPX promotor (*eoCRE* mice) strain to mice with *LoxP*-flanked VAMP-7 alleles (*VAMP-7*^*f/f*^ mice) to generate *VAMP-7*^*f/f*^ *eoCRE* mice (called ‘*eoCRE/V7*’ here). High efficiency *Cre*-mediated *VAMP-7* gene deletion in eosinophils was achieved using an established *eoCRE-loxP* strain associated with the eosinophil-specific EPX promoter^15^. The level of *Cre*-recombined allele was assessed using a four-primer strategy (see Methods section; Fig. 2a). Decreased reporter signals were observed in VAMP-7 null samples, in which the ΔΔCt ratio indicated fewer copies of the target region. Thus, the value of P-a-P-b approaches zero with *Cre*-mediated deletion of ex 3 and 4 of the *VAMP-7* gene (see Methods section for details on primers used in this figure). DNA isolated from white blood cells (WBCs) of a ubiquitous VAMP-7 knockout mice (*Zp3/V7*) served as control; these cells had ΔΔCt values of nearly zero (Fig. 2b). Eosinophils derived from bone marrow progenitors from *VAMP-7*^*f/f*^ mice and heterozygous *eoCRE*^*+/−*^ had higher baseline EPX expression levels than homozygous *eoCRE*^*−/−*^ mice^15^, so heterozygous *eoCRE*^*+/−*^ mice were used as controls in all experiments. Both *VAMP-7*^*f/f*^ and *eoCRE*^*+/−*^ eosinophils had comparable excised:un-excised VAMP-7 DNA ΔΔCt ratios, suggestive of intact *VAMP-7* genes. In contrast, *eoCRE/V7* bone marrow eosinophils exhibited reduced ΔΔCt ratios, suggestive of a VAMP-7 null allele (Fig. 2b). Cytospin analysis revealed that the majority (>90%) of bone marrow cells were eosinophils, while a small percentage (~2–3%) were neutrophils, which accounts for slightly elevated ΔΔCt ratios observed in *eoCRE/V7* bone marrow eosinophils.

To obtain large numbers of eosinophils required for experimental analysis, *eoCRE*^*+/−*^ mice were crossed with eosinophil-overproducing *I5* mice. *I5* mice were also crossed with the *eoCRE/V7* strain (*eoCRE/V7/I5*) to compare with *eoCRE/I5*-derived eosinophils. DNA isolated from eosinophils (CCR3^+^/IL-5 receptor-α subunit [IL-5Rα^+^]/Gr-1^+^/CD4^*−*^/B220^*−*^) and lymphocytes (CCR3^−^/IL-5Rα^*−*^/Gr-1^*−*^/CD4^+^/B220^+^) was used to calculate the percentage of *Cre*-recombined VAMP-7 null allele eosinophils (Fig. 2c). A high percentage (~86%) of *eoCRE/V7/I5* mice-derived peripheral blood eosinophils contained the *Cre*-recombined VAMP-7 null allele, confirming the expression efficiency of the *eoCRE-loxP* system. Traditional PCR validated these findings (Fig. 2d and Supplementary Figure 1b), with the *Cre*-recombined null VAMP-7 allele amplicon exclusively present in eosinophils from *eoCRE/V7/I5* mice.

To augment our molecular analyses, peripheral blood eosinophils from *eoCRE/V7/I5* mice were tested for VAMP-7 protein expression by the western blot. VAMP-7 expression and molecular weight were similar in *eoCRE/I5* eosinophils derived from bone marrow and peripheral blood, as well as human peripheral blood eosinophils (Fig. 2e and Supplementary Figure 1c), while VAMP-7 protein expression was ablated in peripheral blood eosinophils isolated from *eoCRE/V7/I5* mice (96% purity) (Fig. 2e and Supplementary Figure 1c).

The general peripheral blood profiles of *eoCRE*^*+/−*^*, eoCRE/V7*, *I5*, *eoCRE/I5*, *VAMP-7*^*f/f*^*/I5*, and/or *eoCRE/V7/I5* mice were analyzed by differential cell counting (Fig. 2f); no significant differences in eosinophil or other WBC populations were observed between these groups (*p* > 0.05, one-way ANOVA). Eosinophils isolated from peripheral blood of four strains of mice (*I5*, *eoCRE/I5*, *VAMP-7*^*f/f*^*/I5*, and *eoCRE/V7/I5*) were defined and sorted using a combination of cell surface markers and flow cytometry (CCR3^+^/IL-5Rα^+^/Gr-1^mid*−*high^/CD4^−^/B220^−^), and again showed no significant difference in eosinophil numbers (*p* > 0.05, one-way ANOVA; Supplementary Figure 2a).

Growth rates of bone marrow-derived eosinophil progenitors were compared between WT C57BL/6 J, *VAMP-7*^*f/f*^, *eoCRE*^*+/−*^, and *eoCRE/V7* mice (Supplementary Figure 2b). Morphological characteristics, including granularity and granule density, were comparable in eosinophils from *eoCRE*^*+/−*^ and *eoCRE/V7* mice (Fig. 2g). Levels of EPX were equivalent in lung bronchoalveolar lavage (BAL) samples obtained from eosinophil-enriched *I5/hE2/eoCRE*^*+/−*^ and *I5/hE2/eoCRE/V7* mice, suggesting similar EPX expression levels in eosinophils isolated from both strains (Fig. 2h).

### Decreased degranulation in VAMP-7-deficient eosinophils

**Fig. 3:**
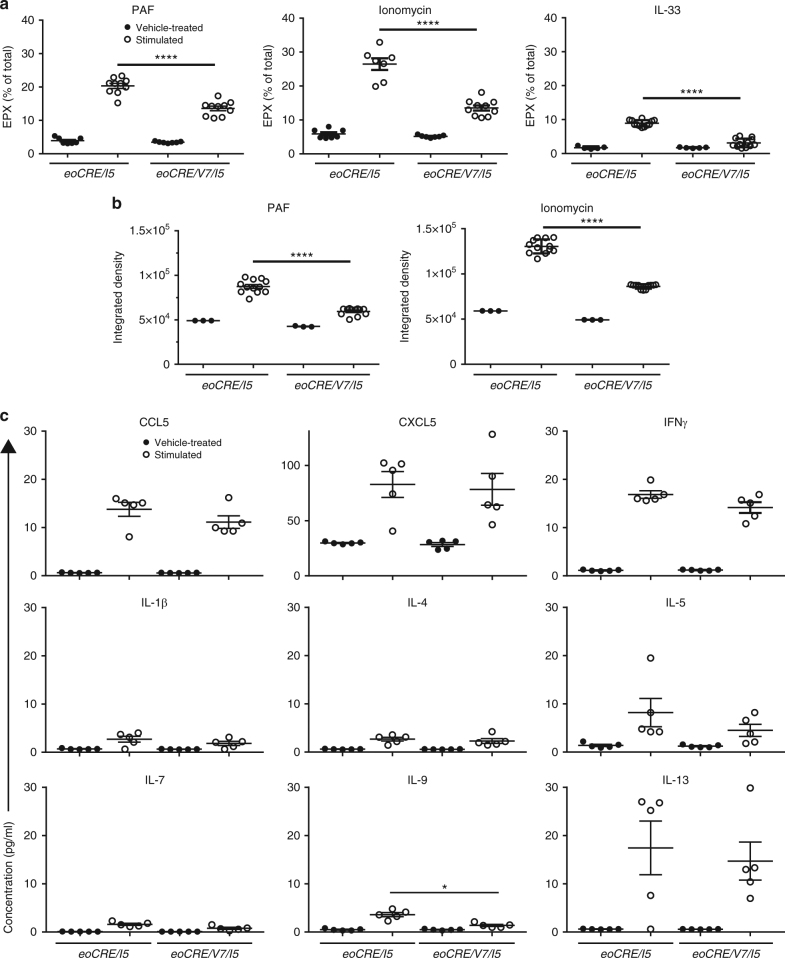
Reduced degranulation responses in eosinophils from *eoCRE/V7* mice. **a** EPX release, calculated as a percentage of total lysed cell EPX content, was determined following PAF (200 ng mL^*−*1^), ionomycin (50 ng mL^*−*1^), and IL-33 (10 ng mL^*−*1^) stimulation of eosinophils isolated from *eoCRE/I5* and *eoCRE/V7/I5* mice. **b** MBP release measured by dot blot analysis as integrated density (×10^4^) of PAF- and ionomycin-stimulated eosinophils isolated from *eoCRE/I5* and *eoCRE/V7/I5* mice. Dimethyl sulfoxide served as vehicle control. **c** Cytokine and chemokine release from *eoCRE/V7/I5* eosinophils. Eosinophils from control (*eoCRE/I5*) and *eoCRE/V7/I5* mice were stimulated for 30 min in the presence of PAF (200 ng mL^*−*1^) and ionomycin (50 ng mL^−1^), and supernatants were assayed for cytokine and chemokine release. Shown are mean ± SEM. All measurements, *n* = 3. *p < 0.05, ****p < 0.0001, using one-way ANOVA with Tukey’s post hoc analysis

To confirm the physiological role of VAMP-7 in mouse eosinophil degranulation, as our previous report suggested a role for VAMP-7 only in streptolysin-*O*-permeabilized human eosinophils^11^, peripheral blood eosinophils were stimulated with PAF (30 min), ionomycin (30 min), or IL-33 (5 h) in vitro. The majority (>90%) of eosinophils remained viable after stimulation with PAF, ionomycin, or IL-33 as measured by Trypan blue exclusion. An in-house-developed EPX ELISA was used to determine extracellular EPX release in supernatants^16^. Eosinophils deficient in VAMP-7 expression (*eoCRE/V7/I5*) showed reduced EPX release in response to stimulation by PAF, ionomycin, or IL-33 (by 33%, 49%, and 65%, respectively) compared with *eoCRE/I5*-derived eosinophils (Fig. 3a).

Supernatants were also evaluated for eosinophil major basic protein (MBP) using a dot blot assay. As seen with EPX, eosinophils released MBP in response to PAF and ionomycin stimulation (Fig. 3b), and amounts of released MBP were significantly lower (*p* < 0.0001, one-way ANOVA) in *eoCRE/V7/I5*-derived eosinophil supernatants compared with *eoCRE/I5*-derived eosinophils.

Finally, we stimulated *eoCRE/I5* and *eoCRE/V7/I5*-derived eosinophils with combined PAF and ionomycin for 30 min, and assessed the release of several cytokines and chemokines in cell supernatants. No significant difference was detected in the release of most cytokines detected (IFN-γ, IL-1β, IL-4, IL-5, IL-7, and IL-13) and two chemokines (CCL11, CXCL5) in *eoCRE/V7/I5* versus *eoCRE/I5* eosinophils (*p* > 0.05, one-way ANOVA). However, levels of the Th2 cytokine IL-9 in *eoCRE/V7/I5* eosinophil supernatants, although very low, were reduced relative to *eoCRE/I5* mice (Fig. 3c).

### Deficient ex vivo degranulation responses in *eoCRE/V7* eosinophils

**Fig. 4:**
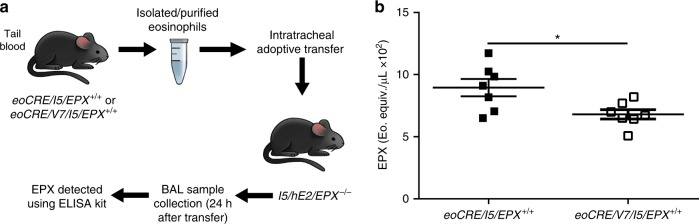
Adoptively transferred *eoCRE/V7/I5/EPX*^*+/+*^ eosinophils in EPX-deficient double transgenic *I5/hE2/EPX*^*−/−*^ mice exhibit diminished degranulation responses. **a** Eosinophils isolated from peripheral blood of *eoCRE/V7* mice adoptively transferred via intratracheal injection into double transgenic *I5/hE2/EPX*^*−/−*^ mice that lack endogenous EPX expression, showed diminished EPX levels in BAL compared to similarly transferred control *eoCRE/I5/EPX*^*+/+*^ eosinophils, determined by EPX ELISA (**b**). Shown are mean ± SEM. All measurements, *n* = 7. *p < 0.05, using unpaired *t-*test

We next evaluated the ability of *eoCRE/V7/I5*-derived eosinophils to release EPX in the lung environment of EPX gene knockout (*EPX*^*−/−*^) mice crossed with the double transgenic *I5/hE2* strain that has constitutive and robust activation of eosinophil degranulation in the airways, leading to a severe asthmatic pathology^17,18^. Given that recipient *I5/hE2/EPX*^*−/−*^ mice lacked endogenous EPX expression, EPX release detected in BALs from recipient mice derived solely from intratracheally transferred peripheral blood eosinophils isolated from *eoCRE/I5/EPX*^*+/+*^ or *eoCRE/V7/I5/EPX*^*+/+*^ mice (Fig. 4a), using our previously reported model^19^. Using this adoptive transfer model, both *eoCRE/I5/EPX*^*+/+*^- and *eoCRE/V7/I5/EPX*^*+/+*^-derived eosinophils exhibited degranulation to endogenous signals in *I5/hE2/EPX*^*−/−*^ mice. However, EPX release from *eoCRE/V7/I5* eosinophils was significantly reduced (~24%, *p* < 0.05, unpaired *t*-test) compared to *eoCRE/I5* eosinophils, suggesting that in vivo activation of VAMP-7-deficient eosinophils was diminished compared with control eosinophils (Fig. 4b).

### OVA treatment to reduced VAMP-7-mediated release of EPX

**Fig. 5:**
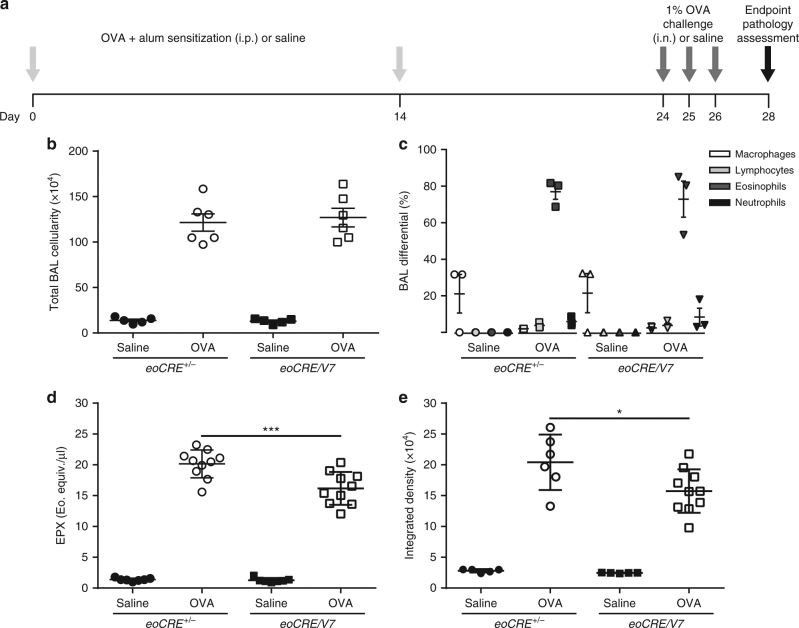
Reduced airway EPX release in *eoCRE/V7* mice following OVA-induced allergic airway inflammation. **a** Schematic timeline of the acute OVA sensitization and challenge procedure used to induce allergic airway inflammation. On day 28, multiple endpoint pathologies were assessed. **b** Total BAL cellularity was compared in saline- and OVA-treated control and *eoCRE/V7* mice, together with (**c**) BAL cell differentials on day 28 of OVA treatment. BAL levels of (**d**) EPX and (**e**) MBP from saline- and OVA-treated control and *eoCRE/V7* mice. Values are mean ± SEM. For experiments shown in Fig. 5b and c, *n* = 5–6. In Fig. 5d and e, *n* = 7–10 BAL samples were measured from individual mice (each marker represents a single mouse). *p < 0.05, ***p < 0.001, using one-way ANOVA with Tukey’s post hoc analysis

To determine the role of VAMP-7 in eosinophil-induced lung pathologies, an acute OVA sensitization and challenge protocol was employed to induce pulmonary inflammation in *eoCRE*^*+/−*^and *eoCRE/*V7 mice. Specifically, *eoCRE*^*+/−*^ controls and *eoCRE/V7* mice were sensitized on days 0 and 14 with intraperitoneal (i.p.)-injected OVA plus alum or saline, then challenged intra-nasally with aerosolized 1% OVA or saline on days 24, 25, and 26 (Fig. 5a). No significant difference in elevated BAL cellularity was observed in OVA-treated *eoCRE*^*+/−*^ and *eoCRE/V7* mice on day 28 (*p* > 0.05, one-way ANOVA; Fig. 5b). Similarly, eosinophil numbers were increased to equivalent levels in both strains upon OVA treatment (Fig. 5c).

Levels of EPX and MBP in BAL samples from these mice were also assessed. While *eoCRE*^*+/−*^ control and *eoCRE/V7* mice injected with saline did not show EPX release into BAL samples, EPX levels in OVA-treated *eoCRE/V7* mice were reduced by ~20% compared to OVA-treated *eoCRE*^*+/−*^ mice (Fig. 5d), and MBP release was reduced by ~23% in *eoCRE/V7* mice (Fig. 5e). These findings show evidence that *VAMP-7* gene deletion led to a reduction of EPX and MBP release in this acute model of allergic airway inflammation.

### VAMP-7-mediated eosinophil degranulation contributes to AHR

**Fig. 6:**
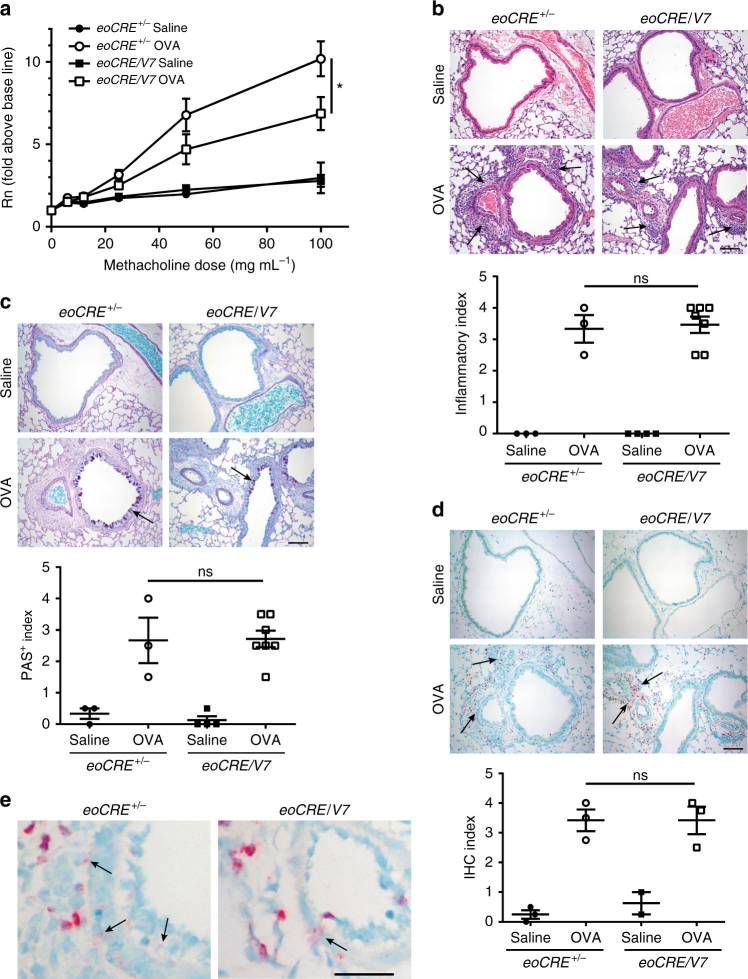
Cell-specific gene deletion of VAMP-7 in eosinophils leads to reduced airway hyperresponsiveness. **a** *eoCRE*^*+/−*^ and *eoCRE/V7* mice (8 weeks old) were subjected to acute OVA or saline (control) treatment, and subsequently challenged with aerosolized methacholine. Airway resistance (Rn) presented as fold above baseline was assessed using an invasive ventilator-based technique (Flexivent). Values are mean ± SEM. All measurements, *n* = 13. *p < 0.05 using ANOVA with Tukey’s post hoc analysis. **b** Representative H&E histological images of lung sections from *eoCRE*^*+/−*^ and *eoCRE/V7* mice subjected to saline or OVA treatment. Arrows indicate regions of inflammation. **c** Assessment of goblet cell metaplasia and airway epithelial cell mucin accumulation by PAS staining of adjacent lung sections to those shown in (**b**). Arrows indicate mucin-producing goblet cells in the large airways of the lungs. **d** Immunohistochemical staining of similar lung sections shown in (**b**) and (**c**) with anti-MBP. Arrows indicate regions of MBP^+^ eosinophilic infiltration. Scale bars, 50 μm. Graphs below each panel of images show quantitative histology, carried out in a randomized, blinded, and unbiased analysis. Data are presented as mean ± SEM of sections of lung measuring ≥200 mm^2^ (3–7 mice per condition). **e** Higher magnification of lung sections stained with anti-MBP from (**d**) to indicate extracellular MBP release. Scale bar, 20 μm. Arrows indicate regions of MBP extracellular deposition resulting from non-cell associated eosinophil degranulation

Eosinophil recruitment and activation has been associated with AHR and airway tissue remodeling in numerous animal models of airway diseases^20^. Consequently, the influence of eosinophil-specific *VAMP-7* gene deficiency in AHR was evaluated following aerosolized methacholine challenge. *eoCRE*^*+/−*^ mice responded to OVA sensitization and challenge as indicated by increased airway resistance (Fig. 6a). However, airway resistance in OVA-treated *eoCRE/V7* mice treated with OVA upon methacholine challenge was significantly lower compared with similarly treated *eoCRE*^*+/−*^ mice (*p* < 0.05, ANOVA; Fig. 6a). These findings show that VAMP-7-mediated eosinophil degranulation contributes to AHR in this model.

Lung sections from *eoCRE*^*+/−*^ mice obtained 48 h after the final OVA challenge showed similar increases in inflammatory infiltrates compared to OVA-treated *eoCRE/V7* mice (Fig. 6b). Increases in periodic acid-Schiff (PAS)-positive, mucin-containing epithelial cells could also be observed in lung sections from OVA-treated *eoCRE*^*+/−*^ and *eoCRE/V7* mice (Fig. 6c). No difference in eosinophil recruitment between *eoCRE*^*+/−*^ and *eoCRE/V7* mice treated with OVA was observed following anti-MBP staining (Fig. 6d), in agreement with our findings for BAL eosinophil numbers following OVA challenge (Fig. 5c). Higher magnification of anti-MBP staining showed a qualitative decrease in extracellular MBP in *eoCRE/V7* mice treated with OVA compared to control, which correlated with our findings for MBP release in BAL samples (Fig. 6e).

Analysis of BAL samples from *eoCRE*^*+/−*^ and *eoCRE/V7* mice for cytokine levels (CCL3, CCL5, CCL11, CXCL1, CXCL10, IFNγ, IL-1β, IL-4, IL-5, IL-6, IL-9, IL12 (p70), IL-13, and TNFα) following OVA treatment showed no changes in any of the cytokines measured, which agrees with the findings for eosinophil cytokine release in vitro (Supplementary Figure 3).

## Discussion

This study demonstrates a contributory role for the R-SNARE, VAMP-7, in mediating physiologically induced release of granule-derived proteins from eosinophils. Previously, VAMP-7-dependent degranulation in eosinophils was only observed in permeabilized human eosinophils activated by intracellular applications of artificial agonists in vitro^11^. Because eosinophils are end-differentiated cells that are difficult to transfect, an understanding of the physiological role of VAMP-7 in receptor-mediated secretion from intact eosinophils could only be achieved using a targeted cell-specific gene knockout model such as the *Cre* recombinase system.

Eosinophil-less animal models have assisted tremendously in gaining insight into the role of eosinophils in the establishment of pathophysiology in allergic airway inflammation^21–23^. However, these models only examine the effect of complete ablation of eosinophils, and do not address unique eosinophil effector functions that are postulated to alter disease outcomes. In this study, we used a more refined eosinophil-specific gene targeting strategy to explore the role of VAMP-7-mediated degranulation in eosinophilic allergic inflammation. Using a combination of a recombination (*Cre*) reporter system and flow cytometry, *Cre*-mediated recombination events could be analyzed at a single-cell level, indicating >95% system efficiency^15^.

Degranulation is defined as the release of granule products by regulated exocytosis or necrosis (cytolysis)^5,6^. A heterogeneous group of granules are located in the cytoplasm of eosinophils, suggesting that eosinophils can undergo regulated exocytosis or piecemeal degranulation^24^. Eosinophil crystalloid granules are involved in classical regulated exocytosis^7^. In addition, a pool of small, rapidly mobilizable secretory vesicles forming part of a tubulovesicular complex, that shuttles proteins from the crystalloid granules to the cell membrane, is associated with piecemeal degranulation^7^. VAMP-7 is a critical component of the vesicular SNARE core complex, and its function is related to regulated exocytosis as well as piecemeal degranulation^11^. In human eosinophils, VAMP-7 was found expressed in secretory vesicles, as well as crystalloid granules, and inhibition of VAMP-7 using a specific antibody in permeabilized cells led to dose-dependent inhibition of EPX and eosinophil-derived neurotoxin (EDN) release, suggesting that VAMP-7 may be involved in all forms of regulated exocytosis in eosinophils, similar to neutrophils^11^.

Exocytosis is a synergistic process that involves numerous intracellular proteins. VAMP-7 deletion in eosinophils diminished but did not completely abolish degranulation at all three levels of biological analysis (in vitro, ex vivo, and in vivo). Incomplete inhibition of granule protein secretion from stimulated *eoCRE/V7*-derived eosinophils suggests that a complex mechanism of selective sorting, transport, and/or fusion exists with a certain level of redundancy. Other R-SNARE proteins may contribute to granule fusion with the plasma membrane, including VAMP-2 and −8, which bind to cognate target Q-SNAREs syntaxin-4 and SNAP-23, expressed in human eosinophils^9,11,14^. Another possible reason for incomplete inhibition of degranulation by *VAMP-7* gene deletion in eosinophils may be related to a separate mechanism of degranulation known as cytolysis in which eosinophils release intact granules following necrosis, a major mechanism occurring in allergic responses^7,25^. However, we found that eosinophil viability was high (≥90%) and eosinophils were intact following stimulation with PAF, ionomycin, and IL-33, suggesting that cytolysis was not a major mechanism of granule protein release in vitro.

Despite the finding that both VAMP-7 and -8 were localized to small secretory vesicles and crystalloid granules in human eosinophils^11^, only VAMP-7 was associated with both EPX and EDN release^11^. VAMP-2, on the other hand, was absent on crystalloid granules, but was found to co-localize with secretory vesicles containing the chemokine CCL5, and traffic to the plasma membrane in response to IFN-γ stimulation^14^. Inhibition of VAMP-2 using a specific antibody in permeabilized cells led to a partial reduction in EPX release but not EDN, suggesting that VAMP-2 is predominantly associated with piecemeal degranulation^14^. The reduction in EPX and MBP release, but not cytokines, from crystalloid granules in *eoCRE/V7*-derived eosinophils implies that a selective VAMP-7-dependent mechanism exists for sorting and mobilization of various granule components during receptor-mediated exocytosis.

The underlying mechanisms that link eosinophil degranulation with allergic AHR are not well understood. Several cationic products of eosinophil granules, including MBP and EPX, have been demonstrated to have cell damaging or activation effects on airway epithelial cells, airway smooth muscle cells, mast cells, and many other airway cells^5^. However, individual gene knockouts of MBP and EPX failed to alter AHR in this allergic model^26,27^. Our findings suggest that complex mechanisms may be involved in multiple granule protein-dependent airway effects that involve additive or synergistic effects of many products of eosinophil degranulation.

Physiological evidence collected in this study indicated that VAMP-7-mediated eosinophil degranulation influences the development of airway immune responses to allergens. AHR was attenuated in *eoCRE/V7* mice compared with controls. However, a recent study has indicated that eosinophil degranulation is not an essential mechanism for the induction of lung pathologies in chronic inflammation^27^, which is in apparent contradiction to our findings. Several reasons could explain the discrepancy in these findings. One is that the cited study examined individual knockouts of MBP or EPX genes, which do not prevent eosinophil degranulation. Eosinophil crystalloid granules contain up to 17 different cytokines and chemokines^28^, as well as many other proteins and enzymes that can alter immune function. An advantage of our present study is that we were able to attenuate both MBP and EPX release without encountering off-target effects resulting from concurrent knockout of MBP and EPX; mainly, the complete ablation of eosinophils from animals^23^. Further studies are warranted to determine the specific contribution of individual eosinophil degranulation products in asthmatic inflammation.

Allergic asthma is intimately associated with the chronicity and increased severity of airway remodeling orchestrated by many cells including eosinophils^1^. Eosinophils manufacture and secrete cytokines and many granule proteins, and these factors are involved in mesenchymal transition and airway epithelial changes; both events are considered to be major driving forces for remodeling. From a clinical perspective, eosinophils are present in ~50% of asthmatic patients^1^. Along with evidence acquired by animal models of allergic airway inflammation, it has been suggested that eosinophil effector functions play an active role in mediating airway inflammation^21,22,29^. Our allergen provocation experiment suggests that VAMP-7-mediated degranulation from eosinophils is important in exacerbation of AHR.

## Methods

### Materials

Antibody to (anti-) VAMP-2 (mouse; 69.1), VAMP-7 (mouse; 158.2), and SNAP-23 (rabbit) were from Synaptic Systems, Goettingen, Germany. Antibody to (anti-) rat CD125 (also known as IL-5Rα; mouse; T21), CCR3-fluorescein isothiocyanate (FITC; mouse; 83101), and phycoerythrin (PE)-conjugated rat Siglec-F (mouse; E50-2440; Siglec-F-PE) mAbs were from BD Pharmingen, Mississauga, ON, Canada. Antibody to (anti-) PE-conjugated Ly-6G (Gr-1-PE) (mouse; RB6-8C5) was from eBioscience, San Diego, CA, USA. Alexa Fluor 700 mouse anti-human CD4 (RPA-T4; Alexa 700-CD4) was from BD Biosciences, Mississauga, ON, Canada. Cy3-conjugated AffiniPure donkey anti-mouse immunoglobulin G (IgG; H + L) was from Jackson ImmunoResearch Laboratories Inc., West Grove, PA, USA. FITC-conjugated mouse anti-human CD63 mAb (MEM-259) was from AbD Serotec, Raleigh, NC, USA. Magnetic cell sorting (MACS) CD45R (B220) (B cells) and CD90.2 (Thy1.2) (T cells; cat. no 130-049-101) MicroBeads were from Miltenyi Biotec, Auburn, CA, USA. Anti-rat IgG biotinylated mouse-adsorbed reagent was from Vector Labs, Burlingame, CA, USA. Streptavidin-alkaline phosphatase and allophycocyanin (APC)-conjugated mouse B220/CD45R mAb (RA3-6B2; APC-B220) were from R&D Systems, Minneapolis, MN, USA. Lee Labs (Mayo Clinic Arizona, Scottsdale, AZ, USA) provided mouse anti-EPX (MM25-429.1.1), biotinylated rat anti-EPX (MM25-82.2.1), biotinylated rat anti-mouse MBP (MM20 220.1.2), and biotinylated rat anti-mouse eosinophil-associated RNases (Ears) (MT3 25.1.1) mAbs^30^. All antibodies were used at 10 μg mL^*−*1^ or 1:100 dilution for all staining or immunoassays. All antibodies were also validated by the Mayo Clinic (Scottsdale, AZ, USA) for in-house use or by the respective commercial sources on mouse proteins using specific ELISAs.

### Mice

All mice (*Mus musculus*) used in this study were bred against the C57BL/6 background for at least eight generations. Mouse strains included C57BL/6J (WT), C57BL/6-transgenic (Tg) IL-5NJ.1638/Lee Labs (*I5*)^17^, and double Tg *IL-5*/human eotaxin-2 (*hE2*) crossed with EPX-deficient mice (*I5/hE2/EPX*^*−/−*^; Lee Labs^19^). The eosinophil-specific B6.129P2-EPX^tm1(*Cre)*^/Lee Labs/Ozgene strain was also used (*eoCRE*, Lee Labs, Mayo Clinic and Ozgene, Bentley DC, WA, Australia^15^, as well as C57BL/6-Tg(*Zp3-*Cre)93Knw/J (*Zp3/V7*, Jackson Laboratory, MA, USA), B6; and 129-Vamp-7^tm1^/RIKEN BRC (*VAMP-7*^*f/f*^, RIKEN BioResource Center, Ibaraki, Japan). *Zp3/V7* refers to nearly complete gene deletion of VAMP-7 based on widespread gene expression of the zona pellucida 3 (*Zp3*) gene. *eoCRE* mice crossed with *VAMP-7*^*f/f*^ mice generated a new strain carrying eosinophils deficient in VAMP-7 (*eoCRE/V7*)^31,32^. Hemizygous experimental *eoCRE* mice (*eoCRE*^+/*−*^) were used as previously described^15^. To elicit substantial numbers of eosinophils required for in vitro and ex vivo experimental analyses, *I5* mice were crossed with *eoCRE*^*+/−*^ or *eoCRE/V7* strains to generate *eoCRE/I5* and *eoCRE/V7/I5* offspring, respectively, which contained abundant circulating peripheral blood eosinophils. In other experiments, *VAMP-7*^*f/f*^ mice were crossed with *I5* mice to generate *VAMP-7*^*f/f*^/*I5* mice. Male and female mice were maintained and bred in ventilated microisolator cages housed in the Mayo Clinic’s pathogen-free (SPF) animal facility. The following mouse strains are available for distribution from the Mayo Clinic: *eoCRE*, *I5* transgenic, *eoCRE/I5*, *eoCRE/V7/I5*, and *I5/hE2*. *VAMP-7*^*f/f*^ are available from Riken, Japan, while the *eoCRE/V7* strain has been discontinued at our site. Progeny of all crosses were viable and fertile, and mice at 8–12 weeks old were used for experiments. Littermates were equally divided for treatments and controls. Samples were allocated into control and experimental groups according to the transgenic background of mouse strains used. A formal randomization tool was not employed. All procedures were performed according to University of Alberta (Edmonton, AB, Canada) and Mayo Clinic’s animal care ethics committee guidelines, and for human eosinophils isolated from voluntary donor blood samples, procedures were approved by the University of Alberta’s human research ethics committee.

### *Cre-loxP* recombination

To overcome problems associated with ubiquitous gene ablation and to address complex phenotypes associated with the loss of a single gene, tissue-specific genetic recombination was achieved by the *eoCRE-loxP* system^15,31^. A cell-specific, characterized promoter (i.e., EPX promoter in eosinophils) was used to drive *Cre* recombinase expression, thereby restricting enzyme expression in eosinophils which are the only cells expressing EPX in mice^15^. Using embryonic stem cell manipulation, the *loxP* sequences were inserted in flanking regions adjacent to the *VAMP-7* gene locus (*VAMP-7*^*f/f*^) mice to generate cell lineage-specific KO mice^32^. The offspring generated from *eoCRE* bred with *VAMP-7*^*f/f*^ mice were designated ‘*eoCRE/V7*’ mice.

### Eosinophil isolation

Eosinophils used in in vitro experiments were isolated at >98% from the peripheral blood of *I5* and *I5*-crossed strains as previously described^17,33^. Briefly, peripheral blood was layered on top of Histopaque 1119 (Sigma-Aldrich) to yield an enriched eosinophil stratum. The eosinophil-containing layer was quickly treated with red blood cell-lysing ice-cold distilled water and washed with 1× PBS prior to isolation. Anti-mouse CD45R (B220)- and CD90 (Thy 1.2)-coated magnetic beads coupled with the MACS system was employed to isolate eosinophils by negative selection according to manufacturer’s instructions (MACSxpress Eosinophil Isolation Kit, Miltenyi Biotec). Human eosinophils were isolated using MACS eosinophil isolation kits (Miltenyi Biotec). Eosinophil population purity was determined by Diff-Quik-stained cytospin samples.

### Immunofluorescence

Immunofluorescence analysis was conducted on adherent mouse eosinophils extracted from the peripheral blood of *I5* mice as described previously^19^. Mouse anti-VAMP-7 (5 μg mL^*−*1^) mAb was added and detected with Cy3-conjugated AffiniPure donkey anti-mouse IgG, followed by FITC-conjugated mouse anti-human CD63 mAb. Immunofluorescence labeling was visualized using a Deltavision OMX microscope (×60 objective; 1.43 NA; Applied Precision, Issaquah, WA, USA) using softWoRx Suite v. 2.0 for deconvolution, with post-acquisition images analyzed using Volocity v. 6.3 to determine co-localization^19^. All images were acquired at 1024 × 1024 pixels, and subsequent image manipulation (brightness, contrast, and cropping) was carried out using Fiji ImageJ v. 1.5 and Adobe Photoshop CC v. 14.0.

### Subcellular fractionation and marker protein analyses

Purified, unstimulated mouse eosinophils were homogenized and resulting organelles separated as reported elsewhere^34^. In brief, purified eosinophils (~5 × 10^7^) were suspended in ice-cold HEPES-buffered sucrose, centrifuged (300×*g*, 8 min, 4 °C), and resuspended in homogenization buffer yielding 10–15 × 10^6^ eosinophils per mL (see Lacy et al.^34^ for buffer content details), and passed 10–20 times through a ball-bearing cell homogenizer (isobiotec precision engineering, Heidelberg, Germany). The postnuclear supernatant (400×*g*, 10 min, 4 °C) was layered onto a linear Nycodenz gradient which then underwent equilibrium density centrifugation (100,000×*g*, 1 h, 4 °C) to obtain 24 × 0.4 mL fractions, stored at −80 °C until use. Fractions were examined for EPX (crystalloid granule marker) and lactate dehydrogenase (cytosolic marker) activities. Western blot analysis of VAMP-7 and SNAP-23 was performed as previously described^11^ using eosinophil lysates (30 µg protein per lane).

### DNA isolation for genotyping

Tail biopsies were cut and digested using a DNeasy Blood and Tissue kit (Qiagen) according to the manufacturer’s instructions. Samples were stored at 4 °C until PCR analysis.

### *Cre* identification by polymerase chain reaction

DNA recovered from tail biopsies was used as a PCR template for genotypying animals that potentially carried the *Cre*-recombined VAMP-7 allele. *Cre*-recombined VAMP-7 null allele-positive animals were identified using a four-primer (P-1, P-2, P-a, and P-b) strategy (Fig. 2a). The amount of product amplified was indicated using the standardized ΔΔCt value generated by the fluorescence intensities of dsDNA resulting from PCR amplification. Primers P-a and P-b were designed based on the sequence between exons (ex) 2 and 3 of the *VAMP-7* gene. The qPCR-amplified product generated using P-a and P-b primers resulted from excision of ex 3 and 4 of the *VAMP-7* gene following *Cre*-mediated recombination while primers P-3 and P-4 were designed based on the sequence within the un-excised region (ex 1). The ΔCt values, defined in equations (1) and (2), of the amplicon were generated by dividing primers P-1 and P-2 or primers P-a and P-b by the constant (primers P-3 and P-4).

1 $$\left( {{\mathrm{P} \hbox{-} 1} - {\mathrm{P} \hbox{-} 2}} \right)/\left. {{\mathrm{P} \hbox{-} 3} - {\mathrm{P} \hbox{-} 4}} \right) = {\mathrm{\Delta Ct}},$$

2 $$\left( {{\mathrm{P} \hbox{-} a} - {\mathrm{P} \hbox{-} b}} \right)/\left. {{\mathrm{P} \hbox{-} 3} - {\mathrm{P} \hbox{-} 4}} \right) = {\mathrm{\Delta Ct}}.$$

Dividing equation (1) by equation (2) provided a ΔΔCt value ratio for each experimental group, as shown in equation (3).

3 $$\frac{{\left( {{\mathrm{P} \hbox{-} 1} - {\mathrm{P} \hbox{-} 2}} \right)/\left. {{\mathrm{P} \hbox{-} 3} - {\mathrm{P} \hbox{-} 4}} \right)}}{{\left( {{\mathrm{P} \hbox{-} a} - {\mathrm{P} \hbox{-} b}} \right)/\left. {{\mathrm{P} \hbox{-} 3} - {\mathrm{P} \hbox{-} 4}} \right)}} = {\mathrm{\Delta \Delta Ct}}.$$

The control (*eoCRE*^*+/−*^) allele (328 bp PCR amplicon) was derived from ex 2 and 3 using P-a and P-b while the *Cre-*recombined allele (326 bp amplicon) was identified using ex 5-derived primers P-a and P-b. Reaction conditions were as follows: 94 °C for 2 min (annealing) followed by 35 cycles at 94 °C for 30 s, 58 °C for 30 s, and 72 °C for 1 min (elongation), and a final extension at 72 °C for 5 min. PCR amplicons were analyzed using a 2% agarose gel electrophoresis coupled with ethidium bromide DNA staining. Primer sequences used for PCR and qPCR in this study are shown in Supplementary Table 1.

### qPCR

Two primers (P-1 and P-2) were designed based on the sequence located immediately up-stream of the first *loxP* site in ex 3 (104 bps). Primers P-3 and P-4 were designed based on the sequence in ex 1 (i.e., the un-excised control region; 54 bps). VAMP-7 excision efficiency was evaluated using the standard ∆∆Ct method and by comparing the qPCR (BioRad My iQ Single Color Real-Time PCR Detection System; BioRad iQ5 software) results from the excised region of VAMP-7 to that of ex 1; the lower the ratio, the more *Cre*-recombined allele present. Reaction conditions were as follows: 95 °C for 10 s (40×), 55 °C for 30 s, 95 °C for 1 min, and 55 °C for 1 min, followed by a melting cure up to 95 °C at 10 s intervals. qPCR data were normalized to *VAMP-7*^*f/f*^ mice. Primer sequences used for qPCR in this study are in Supplementary Table 1.

### Bone marrow isolation and mouse eosinophil derivation

Both femur and tibia were surgically removed from CO_2_-killed mice, and a scalpel was used to remove the epiphyses of long bones. Femurs and tibias were flushed, and bone marrow suspensions were transferred to in vitro derivation was carried out using the procedure described by Dyer et al.^35^. Bone marrow cell cultures were incubated with recombinant murine stem cell factor (100 ng mL^*−*1^, PeproTech, Rocky Hill, NJ, USA) and Flt-3 ligand (100 ng mL^*−*1^, PeproTech).

On day four of culture, part of the cell culture (15 mL) was resuspended in RPMI supplemented with recombinant mouse IL-5 (rmIL-5; 10 ng mL^*−*1^; R&D Systems), and incubated. Prior to incubation, cells were sampled for cytospin and DNA extraction, as well as for PCR identification of the *Cre* recombined allele. An additional sample was removed from cell culture for DNA extraction 2 days later. On day eight, cells were subsampled before the entire culture was resuspended in RPMI supplemented with 10 ng mL^*−*1^ rmIL-5 and returned to the incubator. After cell culture media were renewed on day 10, cells were resuspended for cytospin and DNA extraction, while supernatants were collected for ELISA analysis of EPX. Prior to cell culture termination on day 12, cells were sampled for cytospin, and visual inspection of Diff-Quik-stained cytospin preparations revealed that >90–100% of total cells were eosinophils.

### WBC isolation by red blood cell lysis

Heat lamp-warmed mice were placed in a holder to collect peripheral (tail) blood from animals. Blood added to heparin-containing Eppendorf tubes was inverted, and then filled with lysis buffer (BD Pharm Lyse™, BD Biosciences). After lysis, cell suspensions were centrifuged, and cell pellets resuspended by tapping or by gentle vortexing. This lysis process was repeated for up to 10 min or until a clear WBC pellet was visible. The pellet was washed with PBS-containing 0.25% BSA and 2 mM EDTA.

### Sorting of eosinophils by flow cytometry

Single-cell suspensions were stained with antibodies against CCR3-FITC, PE-Siglec-F, PE-Gr-1, APC-B220, and Alexa 700-CD4 prepared in PBS-containing 0.5% BSA and 2 mM EDTA. Cells were sorted using a FACS Aria Cell Sorter (Becton Dickinson; 488 and 635 nm lasers). Sorted eosinophils were collected as CCR3^+^Siglec-F^+^Gr**-**1^+^B220^*−*^CD4^−^ while sorted lymphocytes were pooled and collected as CCR3^−^Siglec-F^*−*^Gr**-**1^*−*^B220^+^CD4^+^.

### Eosinophil-specific VAMP-7 deletion efficiency

To calculate the percentage of eosinophils possessing the *Cre*-recombined null VAMP-7 allele, DNA (40 ng/sample) was isolated from peripheral blood-derived eosinophils (CCR3^+^/IL-5Rα^+^/Gr-1^mid-high^/CD4^−^/B220^−^) and lymphocytes (CCR3^−^/IL-5Rα^*−*^/Gr-1^*−*^/CD4^+^/B220^+^) sorted from WT, *Zp3/V7*, *eoCRE*^***+/****−*^, *eoCRE/V7* or *eoCRE/V7/I5* mice and subjected to qPCR analysis. Ubiquitous VAMP-7 KO mice (*Zp3/V7*) served as a negative control (∆∆Ct value = 0). All data were normalized to the ∆∆Ct value of the lymphocyte population.

### Transmission electron microscopy

Peripheral blood-derived eosinophils of *eoCRE/I5* and *eoCRE/V7/I5* mice were centrifuged (300×*g*, 8 min, 4 °C), resuspended in Trump’s fixative (1% glutaraldehyde, 4% formaldehyde, and 0.1 M phosphate buffer; pH 7.2), and prepared for transmission electron microscopy as described by earlier studies^26,36^.

### In vitro stimulation of mouse eosinophils

Eosinophils isolated (>98% purity) from the peripheral blood of *eoCRE/I5* and *eoCRE/V7/I5* mice were stimulated in vitro using a procedure described by Dyer et al.^37^. Purified eosinophils were collected by centrifugation, and aliquots were incubated with PAF (200 ng mL^*−*1^; Alexis Biochemicals, Farmingdale, NY, USA)^37^, ionomycin (50 ng mL^*−*1^; Sigma-Aldrich)^38^, IL-33 (10 ng mL^*−*1^; Peprotech) or dimethyl sulfoxide (vehicle control) for 30 min^39^. Aliquots were centrifuged twice to remove debris and supernatants stored at −80 °C until further analysis. In addition, eosinophils from *eoCRE/I5* and *eoCRE/V7/I5* mice were stimulated with PAF (200 ng mL^*−*1^) and ionomycin (50 ng mL^*−*1^) for 30 min, and resulting cell-free supernatants were analyzed using a panel of antibodies recognizing cytokines (IFN-γ, IL-1β, IL-4, IL-5, IL-7, IL-9, and IL-13) and chemokines (CCL5, CXCL5) using a commercially available custom-designed multiplex bead-based assay (Eve Technology, Calgary, AB, Canada).

### Intratracheal transfer of eosinophils into *IL-5/hE2/EPX*^*−/−*^ mice

Purified, ice-rested eosinophils (1 × 10^7^ cells per 25–30 μL PBS) or vehicle control (PBS, 25–30 μL) were installed into the trachea of isoflurane-anaesthetized mice (*IL-5/hE2/EPX*^*−/−*^). BAL samples, collected from recipient mice 24 h post installation, were spun and resulting supernatants centrifuged again (16,000×*g*; 10 min; 4 °C) to remove cellular debris immediately prior to storage at −80 °C until later analysis.

### Granule protein and cytokine/chemokine assays

EPX release was measured using an ELISA kit developed by the Mayo Clinic (Scottsdale, AZ, USA)^16^. Standards for EPX analysis were prepared by purifying eosinophils (14.6 × 10^6^ cells per mL) isolated from the peripheral blood of *I5* mice. After the cell suspensions were spun and their supernatants (200 μL) removed, 250 µL of 0.22% hexadecyltrimethylammonium bromide (Sigma) in 0.3 M sucrose solution was added to lyse cell pellets. Lysates were vortexed (1 min), flash-frozen in liquid nitrogen, and stored at −80 °C. Lysates standards were thawed on ice and pulse-spun (16,000×*g*) immediately prior to use.

The release of MBP was assessed using an immunoblot assay as previously reported^18^. Briefly, 10 μL aliquots of samples were pipetted onto nitrocellulose membranes, which were then dried for 15 min and then incubated with biotinylated rat anti-mouse MBP for 1 h at room temperature, blocked with 1% “Blocker Casein” in PBS (Pierce) for 45 min, and thrice washed with PBS-containing 0.05% Tween 20 prior to incubation with streptavidin-alkaline phosphatase conjugate (Roche Applied Science) for 1 h at room temperature. Membranes subsequently washed with PBS-containing Tween 20 were incubated with Lumi-Phos WB Chemiluminescent Substrate (alkaline phosphatase; Pierce) and chemiluminescence intensity detected using CL-XPosure Film (Pierce). Samples were compared to a protein lysate standard curve derived from known eosinophil numbers.

### Airway inflammation induction by acute OVA sensitization and challenge

Experimental *eoCRE*^*+/−*^ and *eoCRE/V7* mice were sensitized and challenged with chicken OVA according to the procedure described in work published previously^36^. Briefly, male mice were sensitized on days 0 and 14 by i.p. injection of 20 µg OVA (Sigma-Aldrich) and 2.25 mg Imject Alum Adjuvant (Thermo Fisher Scientific) resuspended in 100 µL 0.9% sodium chloride (saline control; Hospira, Saint-Laurent, QC, Canada). On days 24, 25, and 26, sensitized mice were exposed for 25 min to aerosolized (1% w/v) OVA dissolved in saline within individual compartments of a mouse ‘pie’ chamber (Braintree Scientific, MA, USA) using a Pari IS2 nebulizer (Sun Medical Supply, Kansas City, KS, USA) connected to air compressor (30 mg pressure; PulmoAID, DeVilbiss, Somerset, PA, USA) while control mice received an aerosol challenge with saline for the same amount of time. Mice were rested on day 27 and the next day assessed for pulmonary infiltrate, histopathology, and lung function. Several mice were then killed, and BAL collected as described below.

### Preparation and quantification of BAL-derived cells

BAL fluid was collected according to a procedure described by Lee et al.^17^. Briefly, tracheotomy was performed to expose the trachea of sodium pentobarbital-killed mice. After an 18-gauge catheter (1.3 × 30 mm; BD Angiocath, BD Biosciences) was inserted into the trachea, the lungs were lavaged with 1 mL aliquots of ice-cold PBS-containing 0.2% BSA (Thermo Fisher Scientific); 0.7–0.9 mL instilled lavage fluid was typically recovered. Collected BAL samples were centrifuged (500×*g*; 5 min; 4 °C) to pellet cells later used for cytospin and differential cell counts. Lysis buffer (1×; 70 µL; BD Pharm Lyse) and 5% BSA in PBS (500 µL) were added to the cell pellet if undesired red blood cells were present. Total BAL cell counts were carried out using a hemocytometer. All BAL supernatants were centrifuged further (16,000×*g*; 10 min; 4 °C) to remove lung debris and stored at −80 °C until further analysis.

### Cytospin staining for differential cell counts

Samples (~100,000 cells per mL) were loaded onto pre-coated (10% FBS) ColorFrost Plus microscope slides (Thermo Fisher Scientific) and spun (500 rpm; 5 min; RT) in a cytocentrifuge (Thermo Scientific Cytospin 4, A78300003). Air-dried slides were differentially stained using a modified Wright Stain technique (Siemens Diff Quik Stain Kit, Thermo Fisher Scienfic). Then, slides were immersed in xylene and cover-slipped using Shandon Consul-Mount (Thermo Fisher Scientific). Cells (300 cells per sample) were counted to obtain differential cell counts of BAL samples.

### Histopathology and IHC

Lung tissues from *eoCRE*^***+/****−*^ and *eoCRE/V7* mice were inflated in situ with 10% formalin, fixed by 4% (w/v) paraformaldehyde perfusion, embedded in paraffin, and placed onto slides. Histopathological changes of the airways were assessed using conventional staining procedures described elsewhere^17,21^. Briefly, lung sections (4 µm) prepared from formalin-fixed, paraffin-embedded tissue blocks were stained with H&E, Masson’s Trichome or PAS. H&E staining allowed for a general assessment of histopathology including inflammatory cell infiltrates, epithelial and airway smooth muscle hypertrophy, and/or hyperplasia. Masson’s Trichrome facilitated the evaluation of collagen deposition and fibrosis while goblet cell metaplasia and airway mucin accumulation were assessed by H&E staining. Eosinophils were detected by rabbit polyclonal anti-mouse eosinophil MBP antiserum^40^.

IHC of rat anti-mouse MBP mAb stained lung sections (4 μm) have been described elsewhere^41^. Rat anti-mouse MBP mAB (200 µL; 2 µg mL^*−*1^) diluted in 1.5% normal rabbit serum or negative control (Rat IgG, Vector Labs) was added to each slide; unbound antibodies were removed and bound rat anti-mouse MBP mAb was detected by anti-rat IgG biotinylated mouse adsorbed reagent (secondary antibody; 200 µL; 0.4 µg mL^*−*1^; Vector Labs). After appropriate counterstaining, rinsed slides were air-dried and cover-slipped with Shandon Consul-Mount. Our clinical lung pathologist was blinded to all histology during quantification. Slides were provided with a simple numbering identification. The pathologist provided a written report of the findings without knowledge of the groups.

### Assessment of methacholine challenge-induced AHR

Lung function was evaluated 48 h after final OVA challenge using previously described methods^21^. Briefly, tracheotomy was performed on pentobarbital sodium (diluted 1:5 in saline; Abbott Laboratories)-anesthetized mice (i.p. injection; 90 µg per g body weight). A fire-polished, glass endotracheal cannula (18G), inserted into the trachea and secured by sutures, was attached to both a pneumotachograph (model 8410, Hans Rudolph) and an ultrasonic nebulizer (Porta-Sonic model 8500 C, DeVillbiss Health Care) for airflow obstruction with aerosolized methacholine (Sigma-Aldrich). Pancuronium bromide (Sigma-Aldrich)-paralyzed mice (0.5 µg per g body weight) were placed on a 37 °C heating station to maintain body temperature and a calibrated computer-controlled ventilator (Flexivent, SCIREQ, Montreal, QC, Canada; tidal volume: 8 mL per kg; frequency: 2.5 Hz) provided ventilation; the ventilator expiratory line was submerged in water to apply a positive end-expiratory pressure (2–3 cm H_2_O). Increasing concentrations (0, 3, 6, 12, 25, 50, and 100 mg per mL) of aerosolized methacholine in sterile saline was delivered to the animal’s trachea (20 breaths per min; 30 s; tidal volume of ventilator piston: 0.8 mL) to assess airway responses. To re-establish a standard volume, lungs were inflated to total lung capacity (30 cm H_2_O) prior to each aerosol challenge. Seven breaths were collected during regular ventilation or after each aerosol exposure to measure the animal’s baseline airway function and resistance, respectively.

### Statistical analyses

Experiments were repeated independently at least three times. All attempts at replication were successful, and no experiment was found to be irreproducible. GraphPad Prism 7 (GraphPad Software Inc., La Jolla, CA, USA) and Adobe Illustrator were used for statistical analysis and to create graphs, respectively. No data were excluded from analysis. Data are presented as mean ± standard error of the mean (SEM). Data were analyzed for significance using either a one-way analysis of variance (ANOVA) with a Tukey’s multiple comparison test or two-tailed Student’s *t*-test. Significance levels were set at either *p* < 0.05 (*), *p* < 0.01 (**), *p* < 0.001 (***), or *p* < 0.0001 (****). No relevant data existed to perform an accurate power calculation; therefore, sample sizes were not chosen a priori but rather to ensure reproducibility.

### Data availability

The data that support the findings of this study are available from the corresponding author upon reasonable request.

## Electronic supplementary material

Supplementary Information
