# Supplementary material for: Vesicle-associated membrane protein 7-mediated eosinophil degranulation promotes allergic airway inflammation in mice
Source: Commun Biol. 2018 Jun 29;1:83. doi: 10.1038/s42003-018-0081-z (PMC6123774; doi:10.1038/s42003-018-0081-z)
Supplement: Supplementary file 1 — Supplementary Information [file 42003_2018_81_MOESM1_ESM.pdf]

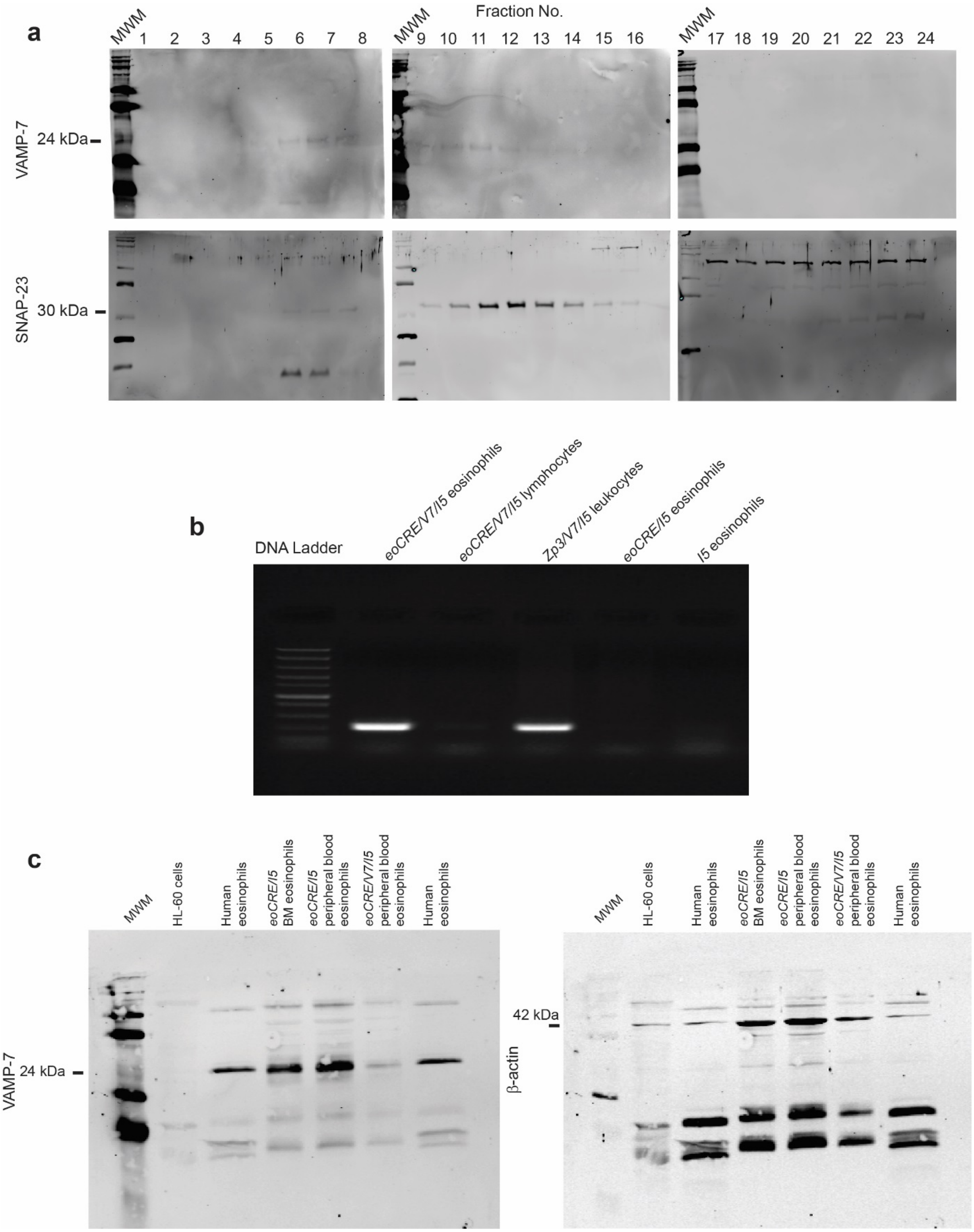

Supplementary Figure 1. Original, non-manipulated representative Western blots and PCR gel. (a) Western blots for Fig. 1b. Total volume loaded from each fraction = 30 μl/lane. (b) PCR gel for Fig. 2d. (c) Western blots for Fig. 2e. Total protein loaded = 30 μg/lane. MWM = molecular weight markers.

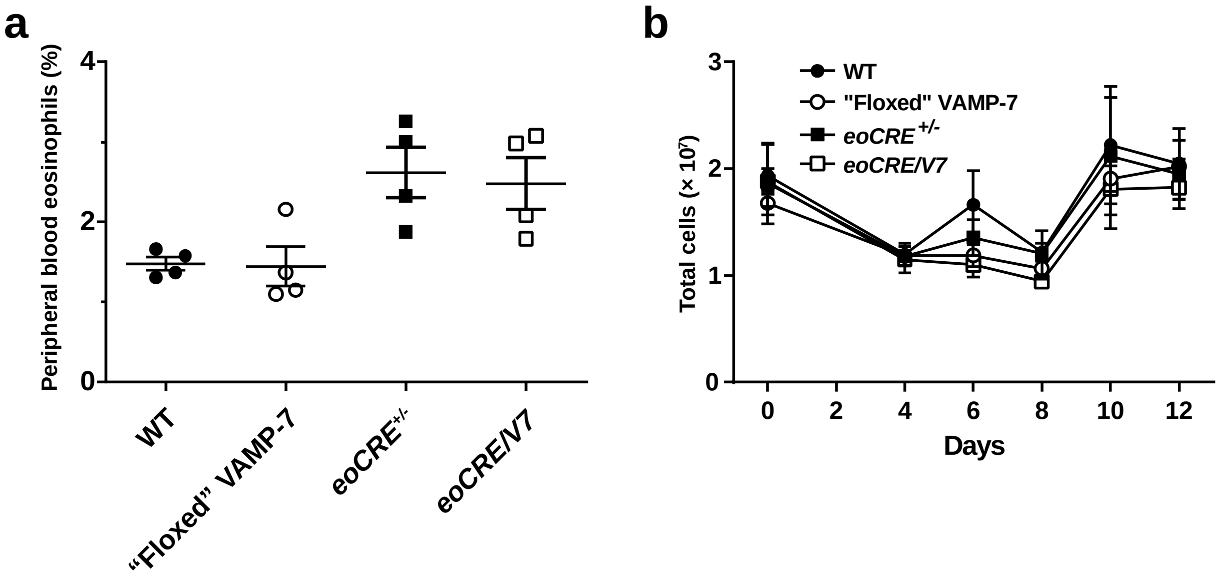

Supplementary Figure 2. Comparison of circulating eosinophil levels, growth curves, and serum EPX levels between control and *eoCRE/V7* mice (a) Peripheral blood-derived eosinophil levels presented as percentage of total WBCs in control and *eoCRE/V7* mice. *n* = 4 individual mice/strain. (b) Growth patterns of *in vitro*-differentiated eosinophils derived from the BM of control and *eoCRE/V7* mice. Total cell counts (~2.0 × 10^7 cells; day 0) for all groups decreased by half (~1.0 × 10^7 cells) during the first four days of culture, due to depletion of non-granulocytic progenitors in the presence of supplementary cytokines (recombinant murine SCF and Flt-3 ligand) in BM culture media. Total cell counts for all groups then recovered (~2.0 × 10^7 cells; days 4-6), likely due to removal of non-granulocytic progenitors and subsequent addition of recombinant murine IL-5, leading to proliferation and maturation of eosinophilic progenitors. Data shown indicate results from 3 independent experiments. Shown are mean ± SEM. In all experiments, $p > 0.05$, using one-way ANOVA with Tukey’s post-hoc test, *n* = 7 individual mice/strain.

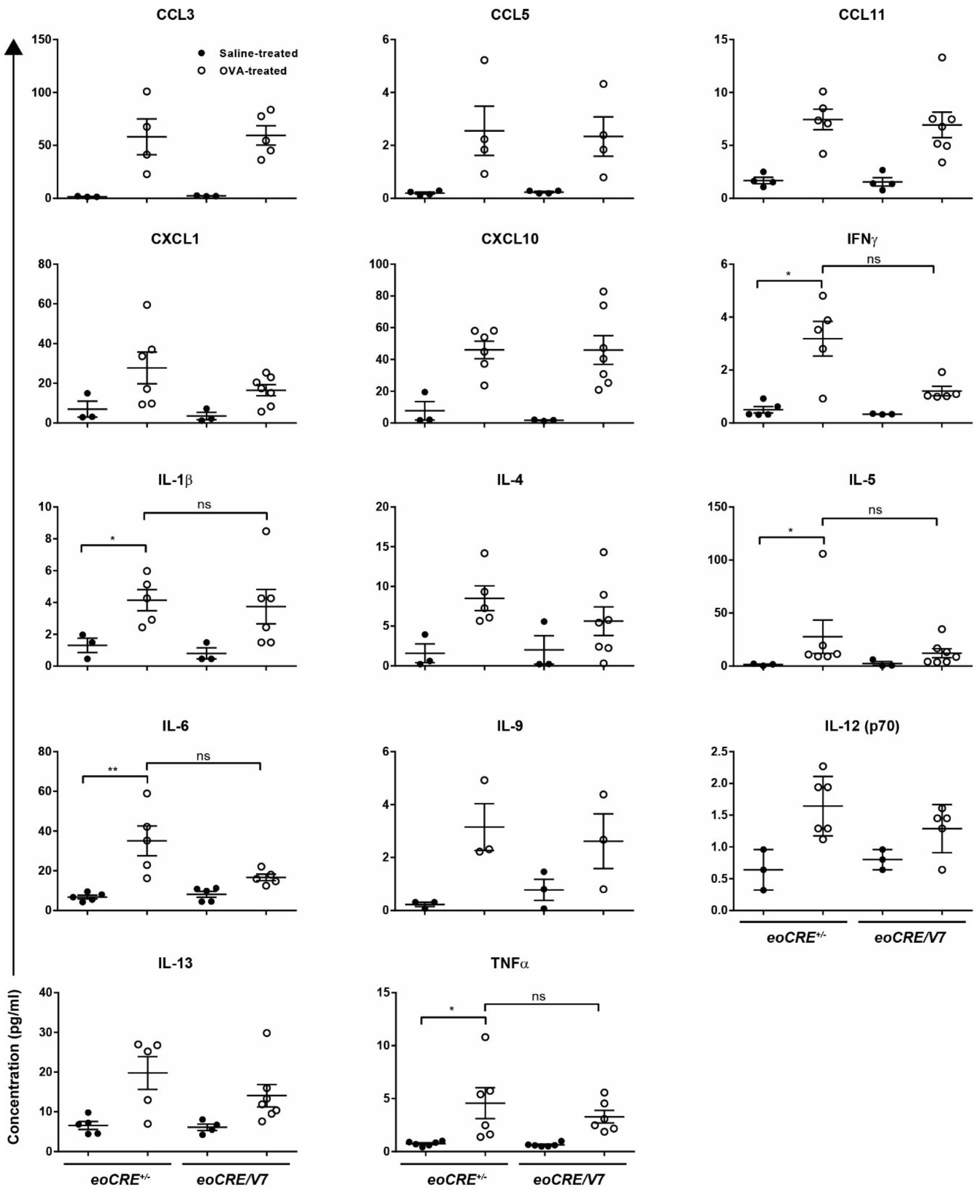

Supplementary Figure 3. Cytokine levels from BAL samples obtained from *eoCRE*$^{+/-}$ and *eoCRE/V7* mice treated with either saline or OVA BAL samples were obtained from *eoCRE*$^{+/-}$ and *eoCRE/V7* mice treated with saline or OVA according to the timeline in Fig. 5a and assessed for cytokine and chemokine levels using a multiplex bead-based assay. Each point represents an individual animal's cytokine level, n = 3-7. * $p < 0.05$, ** $p < 0.01$ using Kruskal Wallis comparison with Dunn's post-hoc test. Panels without statistical comparisons or labelled 'ns' indicate no significant differences.

Supplementary Table 1. List of PCR and qPCR primers used in this study and their sequences.

Name	Sequence (5’ → 3’)
P-a	GCATTACCTGCCCCAGGCAAAACTG
P-b	GAGAGATCAGGGAATTGGTACCGGA
P-1	AACTCCTGGCTGACTCTTTGCATCT
P-2	GGACACAGAGGAAGCAGGTAACGG
P-3	GAAGCTTGCGGCGTCAGGTC
P-4	GGGTCGCCCACTGCCTGAAA
